# Visual search performance is predicted by both prestimulus and poststimulus electrical brain activity

**DOI:** 10.1038/srep37718

**Published:** 2016-11-30

**Authors:** Berry van den Berg, Lawrence G. Appelbaum, Kait Clark, Monicque M. Lorist, Marty G. Woldorff

**Affiliations:** 1 Center for Cognitive Neuroscience, Duke University, Durham, NC 27708, United States; 2 University of Groningen, Univ Med Ctr Groningen, Department of Neuroscience, NL-9713 AW Groningen, The Netherlands; 3 Department of Experimental Psychology, Faculty of Behavioural and Social Sciences, University of Groningen, Groningen, The Netherlands; 4 BCN-NeuroImaging Center, University Medical Center Groningen, University of Groningen, Groningen, The Netherlands; 5 Department of Psychiatry and Behavioral Sciences, Duke University, Durham, NC 27710, United States; 6 School of Psychology, Cardiff University, Cardiff, Wales, CF10 3AT, United Kingdom

## Abstract

An individual’s performance on cognitive and perceptual tasks varies considerably across time and circumstances. We investigated neural mechanisms underlying such performance variability using regression-based analyses to examine trial-by-trial relationships between response times (RTs) and different facets of electrical brain activity. Thirteen participants trained five days on a color-popout visual-search task, with EEG recorded on days one and five. The task was to find a color-popout target ellipse in a briefly presented array of ellipses and discriminate its orientation. Later within a session, better preparatory attention (reflected by less prestimulus Alpha-band oscillatory activity) and better poststimulus early visual responses (reflected by larger sensory N1 waves) correlated with faster RTs. However, N1 amplitudes decreased by half throughout each session, suggesting adoption of a more efficient search strategy within a session. Additionally, fast RTs were preceded by earlier and larger lateralized N2pc waves, reflecting faster and stronger attentional orienting to the targets. Finally, SPCN waves associated with target-orientation discrimination were smaller for fast RTs in the first but not the fifth session, suggesting optimization with practice. Collectively, these results delineate variations in visual search processes that change over an experimental session, while also pointing to cortical mechanisms underlying performance in visual search.

In everyday life humans are constantly exposed to situations in which responding quickly and accurately is important. Hitting a baseball, driving a car, or swatting a mosquito all require clear vision, the appropriate allocation of attention, and the correct response selection to achieve a goal. These abilities are in turn supported by a cascade of neurocognitive processes that must work in conjunction for successful behavior. Factors such as training, learning, fatigue, or lapses of attention affect the efficiency of these processes.

Training, for instance, has been shown to improve information processing^1^,^2^. In our previous paper we focused on the event-related processes that were modulated by training in a visual search task across five consecutive days^3^. Participants were presented with an array of ellipses and asked to find and identify a color-popout target among them and report its orientation. After five days of training, performance improved (i.e., participants became faster at responding without sacrificing accuracy), which was accompanied by training effects on the different phases of the cascade of neural processes, as reflected in series of event related potential (ERP) components elicited by the visual-search arrays. However, a substantial portion of the within-subject variability in response-times (RTs) remained unexplained.

Besides training, there are two other important factors to consider when analyzing RT-performance. One factor is just the variability of performance from trial-to-trial, such as trial-to-trial variations in alertness, attentional task focus, or some other time varying process^4^. A second, related factor is the amount of time performing a task across a contiguous time period (e.g., within an experiment session). For instance, it has been shown that participants’ RTs and brain activity tend to vary across a session^4^,^5^, including Alpha power increases and N1 sensory-evoked ERP responses decreases across session. Such results suggest other changes in information processing that can be due to factors such as within-session learning, mental fatigue, or perhaps simply getting comfortable with the experimental procedures.

To investigate the mechanisms underlying these sources of task performance variation, we examined other facets and relationships of the visual search training data set^3^. In particular, instead of looking at between-session training effects, we explicitly focused on the within-subject RT variability, examining both the fluctuations occurring from trial-to-trial and the changes due to the amount of time the participants had been performing the task within each session. To do so, different neural markers were examined to index changes in the cascade of cognitive processes underlying the within-subject RT-variability.

## Slow-wave CNV activity and oscillatory Alpha as markers for attentional preparation

Part of the within-subject variability in performance seems likely to derive from fluctuations in attentional preparation for each impending stimulus due to factors related to trial-to-trial fluctuations and changes across an experimental session. Attentional preparation might serve as an important predictor of how efficiently one will be able to process the upcoming target and respond to it^6^. Recordings of electrical brain activity provided by electroencephalography (EEG) can serve as a useful method to investigate such attentional fluctuations. Two potential sources of information embedded in the EEG signal that can potentially index fluctuations in preparatory attention are the slow-wave fronto-central contingent negative variation (CNV)^7^ and oscillatory activity in the Alpha (8–14 Hz) frequency range^8^. While the CNV has been used as an index for more task-specific attentional preparation related to the fronto-parietal control network^9^,^10^, Alpha power has been used as an index for both general and selective attentional processes^11^,^12^,^13^, and decreases in Alpha power have been linked to improved target detection and improved visual processing^14^,^15^.

For instance, missing a target in a target detection task^15^, relative to when it was successfully detected, has been associated with higher-amplitude posterior Alpha power prior to the target occurrence. More recently, in a cued Stroop paradigm, preparatory CNV and Alpha activity was linked to attention and RT performance and that these relationships were modulated by motivation^16^. In that study, a cue indicated whether a quick and correct response to an impeding Stroop stimulus could potentially be rewarded or not. The results showed that cue-evoked CNV activity was higher and preparatory Alpha power was lower in amplitude when there was a potential reward. In addition, higher amplitude CNV and lower-amplitude Alpha power also predicted that the response to the upcoming Stroop stimulus would be faster.

## ERP components as markers for visual processing, orientation of attention, and target-feature processing

Performance is not only dependent upon pre-target attentional preparation and alertness, but also on the processing of the target stimulus itself. Visual processing of a stimulus can be indexed by the posterior N1 ERP component (a negative deflection over the posterior channels ~150 ms)^17^,^18^. For instance, in studies which spatially cued participants to direct attention to the potential location of an upcoming visual target stimulus, the N1 was enhanced when spatial attention was present at the location of the target stimulus as compared to when attention was directed elsewhere^17^. It was also found that this N1 enhancement was present when participants had to discriminate the visual stimulus and not when the participants’ task was a simple reaction task. These results show that the N1 can serve as a neural index of visual processing and can be modulated by preparatory attention.

The subsequent reactive orienting of attention towards a lateral target in a visual-search array can be indexed by the hallmark N2pc ERP component^19^. The N2pc, peaking approximately 200 ms after stimulus-array onset, consists of an enhanced negative wave over the occipital cortex contralateral versus ipsilateral to the target stimulus. The further processing of target information (i.e. discrimination of specific features of the target) is reflected in the somewhat later sustained posterior contralateral negativity (SPCN, also known as the contralateral delay activity [CDA]). Previous research of working memory has shown that the amplitude of the SPCN/CDA depends on the demands placed on working memory^20^,^21^. Relatedly, in a visual search task where the size of the search array remained constant but the difficulty in discrimination of the target stimulus increased, the amplitude of the SPCN also increased^22^.

In the present study, we analyzed the relationships between within-subject variability in visual-search RTs and these electrical measures of specific facets of the functional brain activity, with the goal being to gain insight into the neural mechanisms underlying within-subject variability in cognitive task performance.

## Methods

### Participants

Nineteen healthy volunteers (5 female; 18–35 years old) participated in the study. All participants had normal or corrected-to-normal visual acuity and had normal color vision. The experiment was conducted in accordance with protocols that were approved by the Duke Medical Center Institutional Review Board. Written informed consent was obtained from all participants. Participants received 15 dollars per hour in compensation. Data from two participants was excluded due to poor behavioral performance (2 SD below the group mean), and data from another four participants was excluded from the analysis due to excessive EEG noise (mostly artifacts from horizontal eye movements - see EEG preprocessing). Thus, data from a total of 13 subjects were included in the final analysis.

### Task and Stimuli

Stimuli were presented on a 19-inch CRT monitor using Presentation (Neurobehavioral Systems, Albany, CA), with participants seated at a viewing distance of 57 cm. Participants completed five sessions of the visual search paradigm across five consecutive days. Each session consisted of 14 four-minute blocks, each with 140 trials, yielding a total of 1960 trials per session. Participants were given a short break after each block.

**Figure 1:**
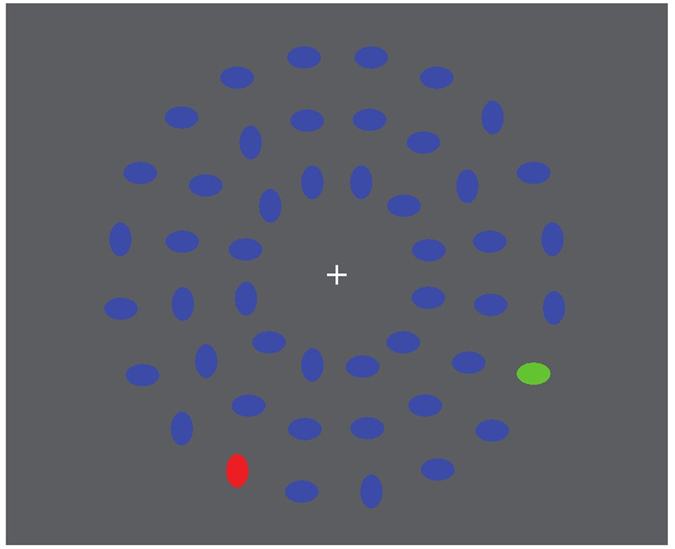
Each search array contained 48 ellipses; 46 of those were in blue, one was green (target) and one red (distractor). The search array remained onscreen for 50 ms, and after a variable ITI (1250–1650 ms) the next search array appeared onscreen.

Each trial consisted of a visual search array, which remained on the screen for 50 ms, and a variable inter-trial-interval (ITI, 1250–1650 ms) (Fig. 1). A white fixation cross remained onscreen during both the visual search array and the ITI. The visual search array consisted of an array of 48 horizontal and vertical ellipses, each subtending a visual angle of ~1.36 × ~0.91 degrees. One ellipse in each array was green (the target popout) and one was red (a non-target popout), with the rest of the ellipses all being blue. Participants were asked to detect the green target ellipse, discriminate its orientation (horizontal or vertical), and indicate the orientation by pressing either the left or right button on a Logitech gamepad using the index finger of the left or right hand.

### EEG recording and preprocessing

EEG was recorded during sessions 1 and 5, using a 64-channel, custom, extended–coverage electrode cap (ElectroCap International, Eaton Ohio). The EEG signals were amplified within the 0.016 to 100 Hz frequency band and each channel was sampled at 500 Hz. During cap application, impedances of all channels was adjusted to below 5 kΩ. Eyeblinks were corrected using independent component analysis (ICA). Prior to the IC decomposition, epochs were extracted from −0.5 to 1.5 s surrounding the presentation of the visual search array. Epochs that contained high levels of noise were excluded from ICA decomposition (using a −150 to 1500 μV threshold detection from which the ocular channels were excluded - the asymmetry of this threshold ensured that most eyeblinks remain in the data). The EEG data were filtered offline using a zero-phase-shift finite-impulse-response filter with 0.5 highpass and 60 hz lowpass filter settings, which were subsequently down-sampled to 250 Hz. Subsequently, independent components (ICs) were extracted using the extended infomax algorithm as implemented in EEGlab13.4.4.b^23^. Finally, all ICs were copied to the original raw data, which was filtered using a zero-phase-shift 60 Hz lowpass filter and subsequently down-sampled to 250 Hz. IC components that reflected eyeblinks (1 or 2 ICs per participant) were removed from the data. Finally epochs were extracted from −2.5 until 2.5 s after onset of the visual search array. Epochs containing any remaining artifacts (horizontal eye movements, muscle noise) were detected using a 110 μV threshold −1.5 to 1.5 s [the threshold was slightly adjusted for some participants] and a 30 μV step function −0.2 to 1 s around the target) and excluded from further analysis.

Frequency decomposition for the oscillatory analysis was performed by means of multiplying the data with a sliding tapered Hanning-window from −1 to 1 s around the onset of the visual search array. The sliding window moved across time with steps of 50 ms. The tapered window had a width of 3 cycles for 3 to 7 Hz, 5 cycles from 8 to 14 Hz and 10 cycles for above 14 Hz for determining power in the theta, alpha and beta band, respectively. Frequency power was estimated by means of a discrete Fourier transform from 2 to 30 Hz with a resolution of 1 Hz. (as implemented in the FieldTrip toolbox^24^). Subsequently, the *natural log* transformed power (P) for every trial (i) was converted for every time (t), frequency (f) and electrode (e) data point to a z-score, across both sessions according to the following equation:


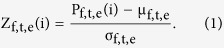


Classically, ERP analysis is done by selecting a subset of trials based on some criteria (e.g., different cognitive conditions, a median split based on RTs) and averaging the corresponding EEG epochs to yield the ERP (or time-locked-averaged EEG signal). However, by discretizing continuous variables (e.g., RTs), one can lose substantial power^25^,^26^. To more fully utilize the continuous nature of RTs across trials, as a final preprocessing step a linear model was run on both the raw EEG and decomposed frequency data in which the dependent variable was either the EEG amplitude in microvolts or the log and z-transformed power. For the predictor variables, first, the RTs and time-in-session were z-transformed for each session separately (z-transformed time-in-session results in the same scale for both sessions). After transforming the data, a linear model was run separately for every subject, session, time, and scalp channel, or in the case of the frequency data every frequency point. The associated design matrix thus had the following specifications: target side (left or right), RTs (z-transformed), and time-in-session (z-transformed trial number). Additionally, interactions between each factor were included in the design matrix. Time-in-session z-transformed values represented the scaled number of visual search trials the participant had performed up to that point within the session.

The estimated beta weights obtained from the linear model, for both the ERP and oscillatory analysis, were used to model the responses for the different conditions. This resulted in the different ERPs_m_ and ERSPs_m_ (event related spectral perturbations) for each subject and condition of interest (subscript “m” stands for “modeled”). To visualize the different conditions we chose the following parameters for time-in-session: early [1.5sd in z-space, corresponding to ~trial 130 within a session] and late [~trial 1830]) and the parameters for RTs: fast [−1.5 sd below the mean of that subject within a session] and slow [1.5 sd above the mean for that subject within a session]). As a result, the final ERPs_m_ or ERSPs_m_ could, for example, represent a *fast response, in the target left condition, early in the first session*. These ERPs_m_ values contain the intercept, and consequently the traditional ERP morphology is maintained, using these modeled values, which is crucial for being able to analyze, visualize and compare these modeled ERPs_m_ responses with standard ERPs in the existing literature. Accordingly, the resulting event-related ERPs_m_ and ERSPs_m_ can be analyzed similarly to a traditional ERP analysis with the advantage of utilizing the continuous nature of time-in-session and RTs^27^,^28^.

To analyze potential preparatory slow-wave CNV activity we estimated a linear slope prior to stimulus onset (−700 to 0 ms) on each trial and each channel. Subsequently we ran the regression model on these slope coefficients. Finally, to analyze the N2pc and SPCN components, the activity in the ipsilateral channels (relative to the target ellipse) was subtracted from the activity in the contralateral channels (relative to the target ellipse), and then was collapsed over target side^19^.

### Statistical Analysis

Behavioral data (RTs and accuracy) were analyzed using repeated-measures ANOVAs. Mean accuracy (correct trials divided by total number of trials), RTs, and variability (SD) were calculated for each bin of 280 trials (i.e. 2 blocks). Occipital Alpha oscillations and the N1, N2pc, and SPCN ERP components were determined in two occipital regions of interest (ROIs) (channels 41, 43, 53, and 55, corresponding to the four sites in our caps nearest to standard sites P07 and O1, and channels 42, 44, 54, 56; corresponding to our four sites nearest to standard sites P08 and O2). Mean amplitudes from the regression-derived ERPs_m_ and ERSPs_m_ were calculated for each condition. Prestimulus Alpha power (8–14 Hz) was measured between −700 ms and stimulus onset. Mean peak amplitudes were calculated for the N1 (136 to 176 ms), the N2pc (200 to 250 ms) and the SPCN (350 to 600 ms). Onset latency of the N2pc was assessed by measuring for each subject and condition the time-point at which the N2pc reached an amplitude of 0.75 uV, which was 50% of the smallest N2pc condition (absolute criterion^29^. We defined a fronto-central ROI (Cz, Fz and their neighboring lateral channels) to measure the CNV.

For statistical analysis of the ERP_m_ and ERSP_m_ data, we defined three factors; first, the session effects (i.e. session 1 vs. session 5), second, the effect of speed (fast vs. slow RTs −1.5 SD above or below the participants mean RT for each session), and finally, the effect of time-in-session (how many visual search trials a participants had performed within a single session, which again was extracted from the model for the activity around trial# 130 and trial# 1830 for early and late, respectively). To test the effects of session, speed, and time-in-session on brain activations, we ran a three-way repeated measures ANOVA with those factors. Together with the repeated-measures ANOVA we reported generalized effect sizes^30^,^31^, 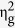. Additionally, repeated-measures t-tests were conducted to interpret significant interactions (p < 0.05).

To examine the relationship between prestimulus Alpha and the posterior N1, we extracted the amplitudes of the prestimulus Alpha and the N1 (according to the time-of-interest and region of interest specified above) from the single trials from each session. Subsequently we extracted the correlation coefficients between prestimulus Alpha and N1 separately for each subject. Finally, we conducted t-tests on the obtained correlation coefficients. All statistical analyses were performed using the statistical programming language R^32^.

## Results

### Behavior

**Figure 2:**
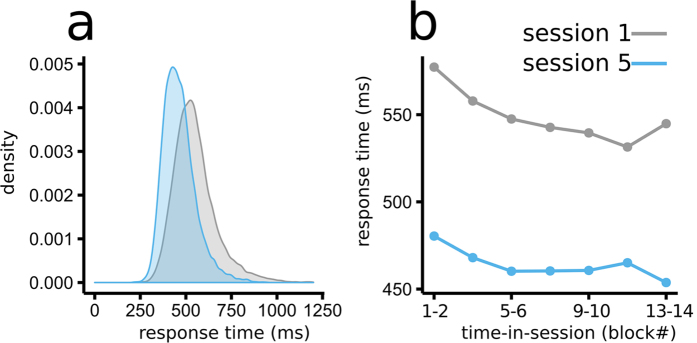
Performance in session 1 and 5. (**a**) Probability density plot showed that there was substantial RT variability within sessions 1 and 5. (**b**) Part of this variability can be explained by an overall decrease in RTs during each session. Each point shows the mean RT for two blocks (280 trials).

As previously reported^3^, participants showed significantly faster RTs in session 5 compared to session 1, while accuracy remained relatively unaffected by training (RTs [SD]: session 1: 554 ms [66] and session 5: 467 ms [53]: F(1, 12) = 99.0, p < 0.001; Accuracy[SD]: session 1: 90% [5.4] and session 5: 91% [6.6]: F(1, 12) = 0.16, n.s.). Closer inspection of the RT distributions (Fig. 2a), however, revealed that, irrespective of the observed performance improvement between sessions, variation in RTs within each of the session remained. Moreover, in both sessions we observed a decrease in RT within a session (time-in-session: (F(1, 12) = 13.0, p = 0.003) (Fig. 2b). The mean variability across subjects in RTs in session 1 was 110 ms, which decreased after the multi-day training to 81 ms in session 5 (F(1, 12) = 72, p < 0.001, 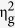 = 0.31). Additionally, the RT variability did not significantly change with time-in-session (F(1, 12) = 0.11, p = n.s., 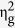 < 0.01). Accuracy remained constant across each session (F(1, 12) = 0.42, n.s.).

### Electrophysiological results

The electrophysiological results and statistics presented here as ERPs_m_ and ERSPs_m_ (modeled ERPs and modeled ERSPs) are all based on the regression-derived coefficients. These regression coefficients were derived separately for each time, channel, and, for the ERSPs_m_, frequency point. Subsequently using these coefficients, including the intercept, we reconstructed ERP_m_ and ERSP_m_ analogues to a classic factorial design that would be derived with conventional selective averaging. The results are visualized for responses that were given near the beginning (trial number 130) or end (trial number 1830) of each session. Additionally, for the RTs, results are based on the estimated neural activity when the participant’s response speed was 1.5 SD above or below its mean separately for each session. These values would correspond to the points on the regression line where ~13.4 percent of the responses were faster or slower than 1.5 SD below or above the mean, respectively. By z-transforming the RTs for every subject and session we removed any effects due to multi-day training, and the reported effects are within-subjects and within-session effects. Although we visualized the extreme responses as those are most interesting to our research question, note that due to the linear modeling, the ERPs_m_ related to the mean RT are identical to the mean ERPs_m_ of the fast and slow responses.

### Prestimulus brain activity

**Figure 3:**
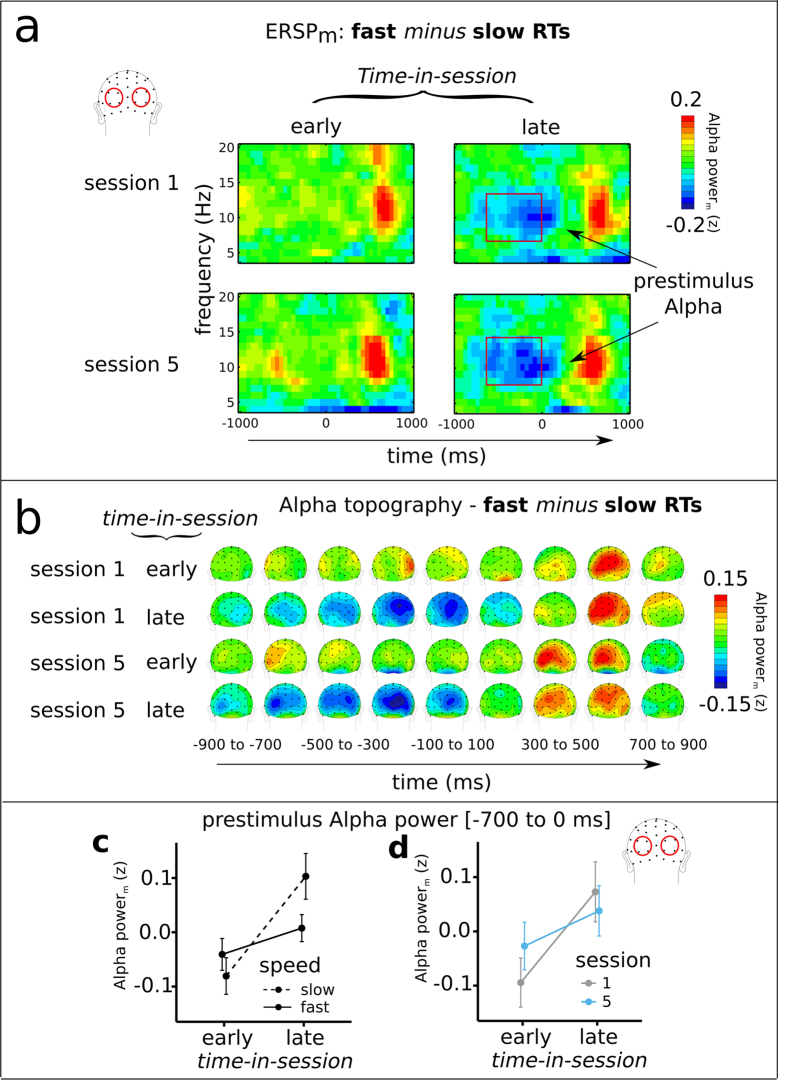
Changes in regression-derived oscillatory power over the occipital channels as a function of RTs. (**a**) Rows depict the different sessions (1 and 5), and columns reflect time-in-session (early and late). (**b**) Topographic maps show that most of the oscillatory effects for activity preceding fast versus slow RTs were over the occipital cortices and late within each session. This pattern of results was similar for session 1 and session 5. (**c**,**d**) Plots reveal the relationship between Alpha power and speed (**c**) and between Alpha power and time-in-session (**d**). Although Alpha power increased significantly throughout session 1, during session 5 this effect was smaller and did not reach significance. (**d**) Within each session, fluctuations in Alpha power had a more profound effect later within the session.

**Figure 4:**
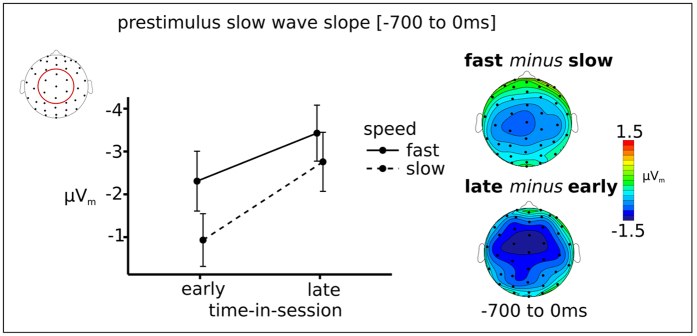
Steeper prestimulus slow-wave CNV activity (700 to 0 ms) preceded fast compared to slow RTs and late vs early in the session.

As noted above, the behavioral analysis indicated substantial within-subject variation in the RTs. We subsequently investigated how slow-wave CNV activity and oscillatory power preceding the presentation of the search array contributed to the RT variability (Figs 3 and 4).

Prestimulus Alpha power was derived from the linear model coefficients of the regression analyses. There was a significant interaction between Alpha power and response speed (time-in-session × speed: F(1, 12) = 10.7, p = 0.006, 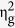 = 0.032) (Fig. 3a,b). Early within a session, Alpha power preceding slow and fast RTs did not differ significantly (t(12) = 1.4, n.s.), while late within a session slower RTs were preceded by higher amplitude pre-stimulus Alpha compared to fast ones (t(12) = −2.38, p = 0.035) (Fig. 3c). This pattern of results suggested that trial-to-trial fluctuations in preparatory Alpha activity were related to RT variability, but this relationship depended upon the amount of time and trials the participant had been performing the task. Important, this relationship between speed and time-in-session did not change after training (speed × time-in-session × session: F(1, 12) = 0.25, n.s., 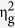 < 0.01). Finally, prestimulus alpha power increased with time-in-session during session 1, but not in session 5 (Fig. 3d: time-in-session × session: F(1, 12) = 5.2, p = 0.046, 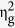 = 0.016; late minus early: session 1: t(12) = 2.4, p = 0.032.; session 5: t(12) = 1.3, n.s.).

The slow-wave CNV analysis (Fig. 4) revealed a negative deflection prior to the onset of the search array (central ROI - mean slope −2.37 μV per 700 ms; F(1, 12) = 17, p = 0.001, 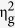 = 0.46), suggesting that there was a CNV-like prestimulus negative deflection. The relationship of the slope of this prestimulus wave with RTs was significant (central ROI - F(1, 12) = 5.6, p = 0.036, 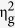 = 0.039) for which faster RTs were preceded by a steeper prestimulus negative slope. Additionally, over the course of the experiment the slope became steeper (central ROI - F(1, 12) = 12, p = 0.005, 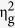 = 0.078). There was no interaction between speed, time-in-session and session.

### Stimulus-evoked sensory-processing activity

**Figure 5:**
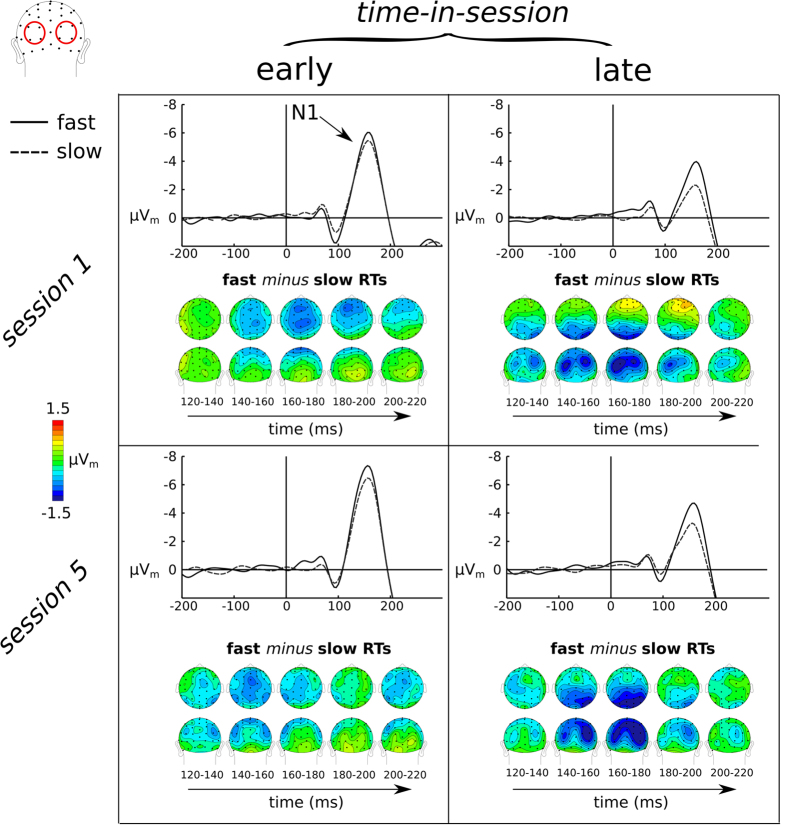
Effect of RT on regression-derived ERP amplitudes (μV_m_) on early visual sensory processing (as reflected by the early sensory-evoked N1 component). Rows depict the different sessions (session 1 and session 5), and columns reflect time-in-session within each session (early and late).

**Figure 6:**
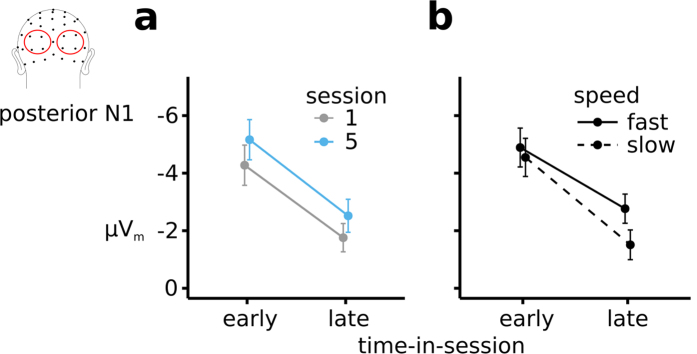
The effects of session number (1 vs. 5), time-in-session (early vs. late) and speed (fast vs. slow) on the posterior N1. The effect of training and time-in-session on the posterior N1 followed distinct patterns (see text).

The ERP_m_ traces and topographic distributions of the posterior N1 (Fig. 5) revealed that, independent of session (session × time-in-session: F(1, 12) = 0.11, p = n.s., 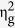 < 0.01), this component dramatically decreased in amplitude over the course of the session (early: −4.7 μV, late: −2.0 μV; time-in-session: F(1, 12) = 47.0, p < 0.0001, 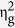 = 0.25) (Fig. 6a). Moreover, we observed that the N1 was more negative (i.e. larger) for fast RTs compared to slow ones (F(1, 12) = 23, p < 0.0001, 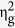 = 0.03), an effect that was stronger later in a session (speed × time-in-session: F(1, 12) = 8.9, p = 0.014, 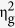 = 0.01, early: t(12) = −2.9, p = 0.01; late: t(12) = −4.2, p = 0.001) (Fig. 6b). The ERP_m_ traces indicated a potential posterior P1 effect for fast versus slow RTs but statistical analysis did not reveal a significant effect (F(1, 12) = 3.1,p = 0.11, 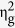 < 0.01).

The relationship between time-in-session and response speed on the N1 was similar to that observed for the slow-wave CNV and prestimulus Alpha power. Accordingly, we examined the relationships between prestimulus Alpha power, slow-wave CNV, and the amplitude of the stimulus-evoked N1. First, we extracted prestimulus alpha power and N1 amplitude on every trial, which we subsequently correlated with each other separately for each subject, and from which we calculated the mean correlation across subjects, revealed that there was an inverse relationship within-subject between prestimulus alpha power and N1 amplitude (mean r = 0.11, t(12) = 4.3, p = 0.001). More precisely, higher amplitude prestimulus alpha power was followed by lower amplitude N1s evoked by the stimulus array. Similarly, the N1 and the slow-wave CNV also correlated (mean r = −0.14, t(12) = −12, p < 0.0001), with steeper prestimulus CNV activity being followed by higher amplitude N1s evoked by the stimulus arrays. Strikingly, there was no observable correlation between the slow-wave CNV amplitude and prestimulus alpha power (mean r = −0.024, t(12) = −1.6, p = 0.14), suggesting separability between the processes these two neural preparatory neural markers reflect.

### Electrophysiological Results: Attentional orienting to the array target

**Figure 7:**
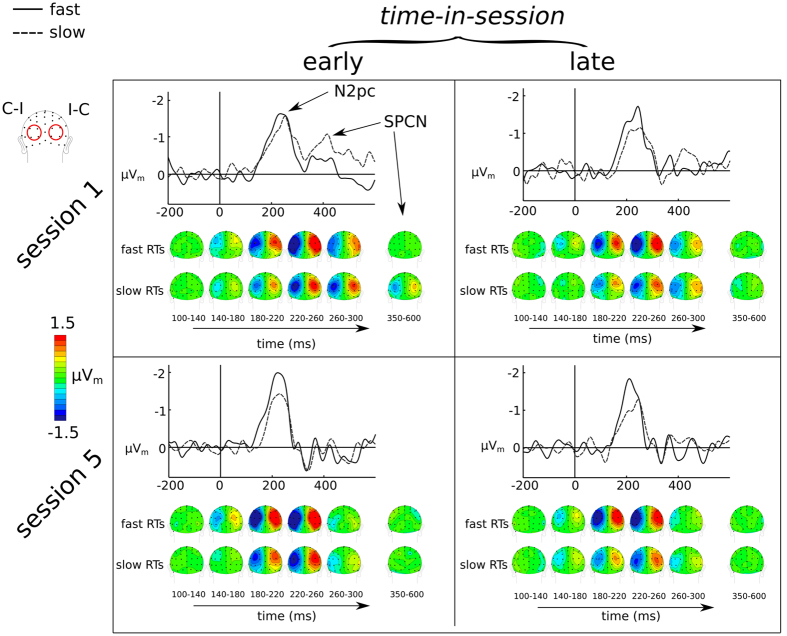
Attentional orienting towards and discrimination of the target in the visual search array. Rows depict the different sessions (1 and 5), and columns reflect where in each session the participant was (early and late). Evoked potentials were derived from subtracting the electrical activity for the electrodes contra- versus ipsilaeral relative to the target location. For the scalp topographies the left channels show the contra-minus-ipsi activity (C-I) while the right channels show the ipsi-minus-contra activity (I-C). Through both sessions, fast RTs were preceded by faster and better attentional orientation.

In addition to being able to investigate effects on the N1 component reflecting early sensory processing, the design of the visual search paradigm allowed us to leverage the lateralized nature of brain activity reflecting the attentional orienting to, and processing of, the target item. In particular, the contra-minus-ipsi lateral analysis showed two attention-related ERP markers of interest: the N2pc and the SPCN, which index the orientation of attention and the further processing of target features, respectively. The regression-derived ERP_m_ waveforms in Fig. 7 showed that the N2pc was larger if the target was followed by fast responses compared to slower ones (effect of speed collapsed across session: F(1, 12) = 16.7, p = 0.0015, 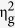 = 0.1). Additionally, the onset of the N2pc was slightly delayed (20 ms), when followed by slower responses compared to fast ones. This, in turn, was reflected by the N2pc amplitude showing a significant earlier difference (165 ms compared to 185 ms after onset of the search array) for fast compared to slow responses (F(1, 12) = 12, p = 0.004, 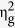 = 0.05).

**Figure 8:**
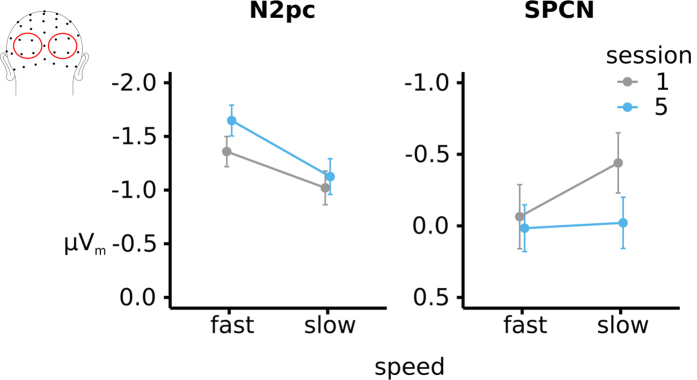
Differential effects for long and short RTs on the N2pc and SPCN between session 1 and session 5. The N2pc was larger in amplitude during session 1 and larger in amplitude when followed by a faster versus a slower response. While, in general, the SPCN was larger in session 1 additionally, throughout session 1, the SPCN was also smaller if it was followed by a faster response compared to a slower one.

The ERPs_m_ for the contra-minus-ipsi analysis as shown in Fig. 7 also extracted the SPCN, which appeared to be larger for slower responses compared to fast ones, at least in session 1. Statistical analysis indeed confirmed that response speed was related to SPCN amplitude throughout session 1, but not in session 5 (session × speed interaction: F(1, 12) = 6.6, p = 0.024, 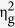 = 0.013, effect of speed session 1: t(12) = 2.9, p = 0.014; effect of speed in session 5: t(12) = 0.3, p = n.s.) (Fig. 8).

In summary, these results showed that both the N2pc and SPCN were related to RT performance: for fast (vs. slow) RTs the N2pc was larger and earlier, throughout both session 1 and session 5. In contrast, the SPCN was smaller for slow versus fast RTs in session 1, but this relationship disappeared after training

## Discussion

In the present study we investigated the cognitive and neural mechanisms that were related to within-subject variability in RT task performance during a visual-search task. For this purpose we examined a number of unexplored aspects of the data set from our visual-search training study^3^. In that study, participants were trained in visual search over five consecutive days, and during the first and fifth session high-temporal-resolution EEG was recorded. The report^3^ focused on the training effects between sessions, finding that training improved performance efficiency and modulated various event-related neural processes. However, there was substantial within-subject variability in the RTs, both before and after training. To investigate the sources of this RT-variability, we examined a set of unexplored relationships between the RT fluctuations and various attention- and perception-related processes. In particular, we investigated how these relationships changed *within* a session (time-in-session), both from trial-to-trial and across the session length, both before and after neural processes were influenced by training.

Key results of the present study showed: (1) Greater attention-related preparatory brain activity (prestimulus Alpha and slow-wave CNV) and early visual sensory processing (N1) preceded fast compared to slow RTs, especially later within a session; (2) Improved efficiency of visual processing later in a session, for both fast and slow RTs (smaller N1 responses); (3) Enhanced attentional orientation (N2pc) to the target also preceded fast compared to slow RTs throughout each session, and (4) Further target-feature discrimination processing (SPCN) differed between fast versus slow RT trials before training, but not after.

First, prestimulus preparatory activity (as reflected in prestimulus negative slow-wave CNV activity and Alpha power) was predictive of response speed. More specifically, steeper negative slow-wave CNV slopes preceded faster RTs throughout the session and independent of training. Additionally, later within each session, higher amplitudes of prestimulus Alpha preceded slower RTs, an effect that was not influenced by training. The observed slow-wave CNV is most likely related to a steeper slope preceding faster RTs indicating better task-specific preparation^33^.

With regard to prestimulus alpha, there has been relatively little research reporting a clear relationship between response speed and prestimulus Alpha power in visual search tasks (or in other tasks more generally). The finding that prestimulus Alpha inversely predicted RTs is in line with previous studies reporting that lower prestimulus Alpha amplitude correlated with subsequent target detection accuracy^15^,^34^. More generally, this finding is accordance with lower alpha being associated with higher levels of general attention and alertness^12^,^35^, here manifesting as an enhanced ability to respond more quickly in performing the visual-search task in an upcoming search array. A possible interpretation for this findings is that Alpha gates the information flow in the visual stream^11^,^36^, and that the relative inhibition of visual regions (as reflected by larger amplitudes of Alpha) slows the ability to process the search-array items in visual cortex.

Similar to fluctuation in preparatory attention, fast versus slow RTs were also preceded by better visual processing, as reflected by a larger posterior N1 sensory component evoked by the array. Additionally, when taking time-in-session into account, this relationship between behavioral response speed and the posterior N1 was greater late compared with early in a session. More specifically, we observed stronger RT effects on the visual N1 particularly later within each session, with larger N1 amplitudes correlating with faster RTs, but to a lesser extent early within a session. Moreover, as with the preparatory Alpha RT effects, the N1-RT effects were larger later within the sessions but did not change after training. This suggests that time-in-session may influence both preparatory attention related to general alertness and visual processing, with both leading to the subsequent effect on RTs. These time-in-session effects, which were quite robust, could in principle be attributed to a range of cognitive processes, such as learning, practice, or fatigue.

In contrast, the CNV-like slow negative wave-RT effects did not change across the session, which as suggested by previous studies^13^,^16^ further confirms that Alpha and CNV components most likely reflect different cognitive processes. As mentioned above, fluctuations in preparatory attention can potentially be viewed as due to a combination of factors such as motivation, mental fatigue, and perhaps others. We found that the markers for preparatory attention, the slow-wave CNV and Alpha, did indeed covary with RT speed. However, whereas the covariation of alpha with RTs changed quite dramatically over time, the covariation between RT and CNV did not. One possibility might be that Alpha power reflects changes in general factors affecting task performance, such as fatigue, motivation, and overall alertness. The CNV, however, potentially comes from the fronto-parietal control network that has been implicated more in task-specific attention. Therefore, the CNV might reflect task specific modulations of attention, in which fluctuations would be a proxy for focusing selectively on the task (versus, for example, thinking about other matters, such as making a grocery list). To further elucidate these effects of time-in-session, we looked at which other neural processes changed as a function of time-in-session.

Another major result was that as a function of time-in-session, collapsed over slow and fast RTs, we observed increases in slow-wave CNV, increased preparatory Alpha power over session 1 and a smaller posterior N1 over both session 1 and session 5. Additionally, prestimulus Alpha and the posterior N1 correlated inversely with one another, in line with what has previously been reported^37^,^38^. We propose several possible accounts for these time-in-session modulations of neural effects: (1) the decrease in the N1 might be related to some sort of neural-response adaptation over time; (2) it might be related to a change in strategy or mental fatigue in how the visual search array is processed over time, or (3) it might reflect a combination of these. There is previous evidence that neural adaptation effects can occur over time; more specifically, after repeated stimulation of the same neural populations, those populations get less and less sensitive, resulting in reduced responses^39^. These are not necessarily permanent neural changes that we refer to as reflecting lasting learning effects, but are more related to repeated stimulation within a certain time interval. However, the changes observed here are not over a mere few seconds or even minutes but occur over a period of an hour (the length of a session). Accordingly, the adaptation explanation would not seem so likely, but it still could be a contributing factor for the observed effects.

The second possible account for the increase in alpha power and decrease in the visual N1 across a session, particularly perhaps for session 1, is that these effects could reflect a change in strategy as to how participants prepare for the upcoming visual search array, which then also results in fluctuations in the RTs. During a session, participants may try to leverage the amount of information to be processed and the subsequent cognitive load^40^,^41^,^42^. If indeed they adopt a different strategy aimed at reducing the information load by more efficient attentional preparation, this might result in faster overall RTs. In other words, by employing a more efficient focusing of attention towards features that are important for task performance, and additionally ignoring and inhibiting task-irrelevant features better, performance efficiency might increase.

Neurally the shift in strategy can be reflected by the prestimulus Alpha and slow-wave CNV effects on RTs, which can be related to a shift in strategy within a session to achieve a balance in the selection of information. More specifically, on the one hand, if information selection is too liberal, it would tend to result in a high cognitive load, which can result in turn in a slow RT. On the other hand, information selection being too strict, and thus inhibiting important information, could also result in a slow RT. Hence, especially later within a session (when a more optimal strategy has been adopted), the balance between too much inhibition (=slow RT) and too little (=high cognitive load) may become apparent by a stronger correlation between prestimulus Alpha and RTs. In other words, by employing a more efficient focusing of attention towards features that are important for task performance, and additionally ignoring and inhibiting task irrelevant features better, performance efficiency might increase. These task-specific fluctuations in preparatory attention might be indexed by slow-wave CNV fluctuations, presumably originating from the fronto-parietal control network, that increased over the course of the experiment.

Additional evidence for this hypothesis comes from the observed modulations of the posterior N1; late within a session the posterior N1 was much decreased in size, and yet participants were able to respond faster as compared to earlier in the session. If the N1 reflects the amount of cognitive effort exerted during the processing of a visual search array, then the N1 decrease would suggest that visual search was becoming more efficient over the course of a session. Importantly, the stronger relationship between response speed and the N1 later within a session suggests that even though visual search has become more efficient there is a tradeoff between visual search efficiency and RTs. Part of improved efficiency can potentially be explained by a more efficient task-specific preparation. Additionally, however, fluctuations in alpha and the N1 might reflect more general changes in motivation, mental fatigue, or learning, which would be less task specific and seem to have a more pronounced influence on RTs later in the session.

Following attentional preparation and the visual processing of the search array, participants needed to direct their attention to the relevant target item to further process its features (here, to discriminate its orientation). On a neural level the larger negativity contralateral to the target side reflects the lateralized attentional allocation with high temporal resolution^19^. The amplitude and latency of the N2pc was closely related to RT-performance, being larger and earlier on trials with fast RTs. Interestingly, even though after training participants had significantly accelerated and enhanced the allocation of attention towards the target, there was still trial-to-trial variability in this process. Even though training can accelerate the allocation of attention on average, it cannot optimize the attentional orienting process to such an extent that it no longer has any variability. In other words, even after extensive training, there is still variation in the allocation of spatial attention from trial-to-trial, which then can ramify into faster or slower RTs, perhaps as a consequence of fluctuations in preparatory attention and visual processing.

Lastly, in untrained participants we found that fast RTs were preceded by a smaller SPCN compared to slow RTs. While the N2pc is a component associated with the orienting and focusing of attention on the target item, the SPCN is of larger amplitude when participants are asked to also discriminate a specific additional feature of the target item^22^. Accordingly, we infer that the SPCN effect observed in session 1 reflects that the further task-relevant feature analysis of the target (i.e. here, its orientation) was more efficient for fast compared to slow RTs. After training, however, the difference in SPCN size preceding slow and fast RTs disappeared. Thus, it might be that this automation reflects the improved maintenance of the memory trace for the target feature (its orientation) necessary to select an appropriate motor response, similar to our interpretation of the shift in strategy with respect to the N1. Either way, the results supports the idea that the processes involved in identification and response to features of the target can be optimized to rely on less information while maintaining the same level of performance. In other words, training can rendering certain processes to become more automatic and effortless^43^,^44^.

Training revealed an increase in the visual N1^3^, suggesting improved visual processing over sessions. In the present study we found that, within-session, for both slow and fast RTs the efficiency of visual processing improved. Participants required less visual processing (smaller N1) of the entire array for the same response time late as compared with early in the session. One interpretation would be that, on the one hand, improved visual processing by training does indeed help subsequently responding fast by recruiting more synchronized neural activity for visual search. On the other hand, however, over the course of the experiment, efficiency could improve, perhaps due to better, more specific preparatory slow-wave processes that benefit visual processing. If true, then a remaining question for future research is why the improved task-specific preparation, and with it efficiency of visual processing, were not transferred by the multi-day training.

In the present study, measures of electrical brain activity were used to provide neural indices for several key attention- and perception-related processes during a visual-search task. By examining how these brain activations covaried with RTs, our data help elucidate our understanding of variations in cognitive task performance in several ways. First, we identified that better preparatory attention and visual processing in visual search tasks – as marked by lower levels of prestimulus Alpha, steeper prestimulus slow-wave CNV slopes, and higher amplitudes of the visual processing related ERP components – lead to faster RTs. Importantly, these variations did not significantly differ as a function of training and were especially apparent later within a session. Additionally, allocation of attention towards the target item in the search array (reflected by the N2pc) also contributed to explaining RT variability throughout each session, independent of time-in-session and training. Finally, further discrimination and responding to task-relevant target features (reflected by the SPCN) seemed to be optimized by training and thus did not play much of a role in explaining RT variability after training. Together, these results provide new insight into how various neural processes during visual search are related on a trial-by-trial basis to task performance, and how and which of these processes can be influenced by other key factors, such as previous training and the length of time spent in one continuous session performing a task.

## Additional Information

**How to cite this article**: van den Berg, B. *et al*. Visual search performance is predicted by both prestimulus and poststimulus electrical brain activity. *Sci. Rep.* **6**, 37718; doi: 10.1038/srep37718 (2016).

**Publisher's note:** Springer Nature remains neutral with regard to jurisdictional claims in published maps and institutional affiliations.
